# Principal component analysis for body condition of Anatolian water buffaloes

**DOI:** 10.5194/aab-69-633-2026

**Published:** 2026-11-09

**Authors:** Onur Şahin, İsa Yılmaz, Ali Kaygısız

**Affiliations:** 1 Department of Animal Science and Technologies, Muş Alparslan University, Muş, 49250, Türkiye; 2 Department of Animal Science, Kahramanmaraş Sütçü İmam University, Kahramanmaraş, 45050, Türkiye

## Abstract

This study evaluated eight body condition traits, which included backbone, ribs, short ribs, hip bone, rump, pin bone, tailhead, and thigh, in 614 Anatolian buffalo cows from 122 farms to investigate underlying structural dimensions using principal component analysis. Body condition was assessed on a scale from 1 to 9. Descriptive statistics showed that backbone had the highest mean score (6.12), whereas tailhead had the lowest (4.92), indicating that the vertebral region is a key determinant of body condition. Trait variability was moderate, with standard deviations ranging from 0.72 to 0.94, reflecting a generally consistent dataset. The factorability of the data was confirmed by the Kaiser–Meyer–Olkin measure (0.892) and Bartlett's test of sphericity (3896.44, p<0.001 ). Principal component analysis revealed a two-factor structure that explained 79.67 % of the total variance. The first factor predominantly represented vertebral and posterior body regions, and the second factor reflected pelvic and lower-body regions. Varimax rotation clarified the factor loadings, with some variables, such as hip bone and rump, loading significantly on both factors, indicating their association with multiple anatomical regions. Phenotypic correlations among traits were positive and significant, highlighting coordinated growth patterns. These findings suggest that body condition traits in buffaloes can be effectively summarized by two main structural dimensions and may facilitate the interpretation of phenotypic variation among Anatolian water buffaloes. The identified principal components provide a useful basis for further research on the potential application of multivariate body condition assessment in these populations.

## 1 Introduction

As is the case worldwide, buffaloes (*Bubalus bubalis*) are raised in Türkiye for milk, meat, and draft power. Recent studies have shown that Anatolian buffaloes are as productive as cattle in live weight gain and feeding performance. The Anatolian buffalo bred in Türkiye originates from the Mediterranean buffalo, which belongs to the river buffalo group (Soysal, 2019; Şahin, 2015; Yılmaz et al., 2021).

Anatolian buffalo herds in Türkiye are raised using extensive grazing systems or semi-intensive systems that combine grazing and barn breeding. In water buffalo breeding in Türkiye, milk yield has been more prominent than meat and draft power. Buffalo milk is used as a raw material for yogurt, butter, and clotted cream, and buffalo meat is widely used in processed meat products, especially sausage.

Body condition reflects an animal's energy reserves at any stage of the production process. It provides useful information about overall health, productivity, the adequacy of the previous feeding program, and planning nutritional requirements for future milk and reproductive performance. To accurately assess body condition, it is necessary to visually inspect and palpate fat reserves in different parts of the body (Şahin, 2011). For this purpose, the cow's spine, ribs, short bones, pelvis, rump, hock, tailhead, and hip area are evaluated by touch and visual inspection on a scale ranging from 1 to 9 or 1 to 5. The evaluation results for each body part are added and divided by 8 to calculate the cow's overall body condition score (DairyNZ, 2021).

The body condition of Anatolian water buffaloes, which is poor at the start of the grazing season, improves from the middle of the season onward. However, calving, which begins at the end of winter, increases at the start of the grazing season. Poor body condition at calving leads to decreased milk yield and fertility problems. Cows entering the calving period with excessive body fat experience more difficult births and metabolic problems (Şahin, 2011). In cows that are excessively fat in the late stages of pregnancy, free fatty acids can be mobilized due to insulin resistance, and a decrease in dry matter intake is observed due to a reduced appetite. For these reasons, the body condition of the cow is very important in the late stages of pregnancy and at calving (Singh et al., 2017).

Therefore, it is crucial that female cattle and water buffaloes begin the calving period in good physical condition. For this reason, cows must be assessed for body condition during the calving period (first week), peak milk production (45th d), end of early lactation (90th d), mid-lactation (150th d), late lactation (200th d), and the late dry period (eighth month of pregnancy).

To summarize similar research on body condition in buffaloes, Alapati et al. (2010) and Saludes et al. (2021) reported average body condition scores of 3.29 and 3.97, respectively, in studies of the Murrah breed. Arote and Siddiqui (2020), who studied changes in body condition at different lactation stages in the same breed, reported scores of 2.93 for early lactation (0–90 d), 2.95 for mid-lactation (91–180 d), and 3.48 for late lactation ( >180 d). Tariq et al. (2012) and Mirza et al. (2013) conducted studies on the Nili-Ravi breed and reported average body condition scores of 3.80 and 4.92 (on a 1–9 scale), respectively. Lubis et al. (2010) reported an average body condition score of 2.90 in their study of swamp buffalo.

Principal component analysis (PCA) is a statistical technique that reduces the number of observed variables to a smaller set of composite variables that explain the maximum variance in the original variables. By reducing the dimensionality of multivariate data, PCA produces new variables that capture most of the variation with a small number of components, which can be used instead of the original variables (Gandhi, 2015; Vohra et al., 2015). In this way, PCA simplifies the analysis of complex, high-dimensional datasets and facilitates model interpretation by separating high-dimensional data into its most explanatory components (Hasan and Abdulazeez, 2021; Vinutha et al., 2023).

Whereas factor analysis focuses on the common variance among observed variables, PCA considers both the total variance of all variables and the variance of each individual variable. Factor analysis reduces the number of original variables to a limited set of unobservable variables, using the relationships among observed variables to explain their interrelationships. It assumes that the variance of each resulting variable represents a significant portion of the total variance. In contrast, PCA uses a minimum number of variables to explain the maximum portion of the variance. This technique assumes that the variance in each representative variable accounts for only a small portion of the total variance (Sadek et al., 2006).

A PCA was performed on 18 morphometric characteristics of male swamp buffaloes (*Bubalus bubalis*) from two age groups (2–3.5 and 3.5–4 years). The study identified five principal components (PCs) of morphometric traits, accounting for approximately 70 % of the total variance in both age groups. It concluded that withers height, chest depth, and hip height could serve as morphometric selection criteria for 3–4-year-old male swamp buffaloes (Putra and Hilmawan, 2022).

In a study conducted at the Swiss Federal Institute of Technology's Chamau research farm on body condition score (BCS), milk yield (MY), milking speed (MS), dry matter intake (DMI), and body live weight (BW) of dairy cows with multiple births, more than 75 % of the total variance for all traits was explained by the first four main components (BCS, MY, MS, and DMI) (Karacaören and Kadarmideen, 2008).

In Indonesia, PCA was used to identify the body measurements and indices that determine body weight in Katjang female goats. PC1 (face length, face width, and face height) accounted for 64.89 % of the total variance, whereas PC2 (cephalic index, length index, depth index, and body index) accounted for 86.84 % of the total variance (Putra and Ilham, 2019).

Domecq et al. (1997) incorporated PCA into a multiple regression model to summarize correlated body condition variables and reduce multicollinearity among predictors. Their study showed that PCA is useful for reducing the dimensionality of body condition measures while preserving their underlying information.

This study aims to reveal the structural relationships among morphological characteristics that determine body condition in Anatolian water buffalo, to analyze the body condition of the evaluated individuals more objectively and comprehensively, and to identify the fundamental components underlying these variables by applying factor analysis to measurements from various body regions. The study seeks to contribute to the development of a simpler, more meaningful, and structurally valid system for assessing body conformation. To the best of our knowledge, this study is the first study applying PCA to body condition traits in Anatolian water buffaloes.

## 2 Materials and methods

The study material included condition scores for eight body parts and overall body condition scores for 614 buffalo cows raised on 122 Anatolian water buffalo farms. To eliminate the influence of different evaluators, the body condition of Anatolian water buffaloes was scored by a single expert in the field. Because records are not kept on farms, factors such as lactation order, lactation stage, age, and milk yield were not considered in the analysis. Microsoft Excel was used to organize body condition data. SPSS version 25.0 was used for all statistical analyses. Principal component analysis was conducted to identify the characteristics that explain most of the variation in body condition.

The PCA technique replaces a set of correlated variables with a smaller set of uncorrelated variables. These variables, called principal components, are linear combinations of the observed variables. This linear combination explains the most variance in the original variables (Truxillo, 2003).

1 $$\mathrm{PC}1=a_{1}X_{1}+a_{2}X_{2}+\ldots +a_{n}X_{n}$$

The varimax rotation in this analysis was performed by rotating the principal components to achieve a simpler structure. The Kaiser–Meyer–Olkin (KMO) measure of sampling adequacy and Bartlett's test of sphericity were used to assess the dataset's validity at the 1 % significance level. The KMO test evaluates whether the variables are suitable for factor analysis based on the correlation matrix. The KMO value ranges from 0.0 to 1.0, with values below 0.5 indicating that factor analysis is not appropriate.

Principal component analysis was applied to buffalo cows using eight variables for body condition scoring: backbone (BB), ribs (RB), short ribs (RS), hip bone (HB), rump (RP), pine bone (PB), tailhead (TL), and thigh (TH). These body condition variables are scored on a scale from 1 to 9. On this scale, scores of 1 to 3 indicate poor body condition, 4 to 6 indicate average body condition, and 7 to 9 indicate fat body condition (DairyNZ, 2021) (Table 1).

**Table 1:** Variations in body condition parameters on a scale of 1–9 (Şahin et al., 2023).

Variables	1–3 (thin)	4–6 (moderate)	7–9 (fat)
Backbone (BB)	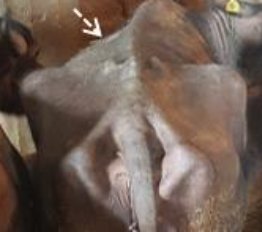	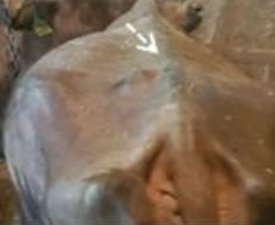	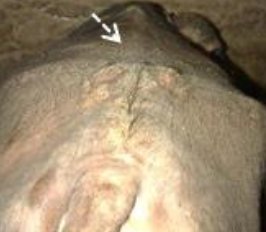
Ribs (RB)	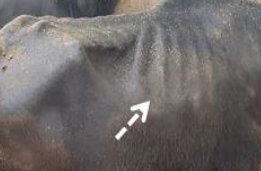	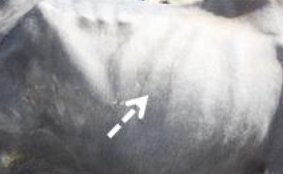	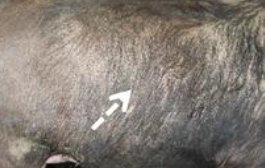
Short ribs (SR)	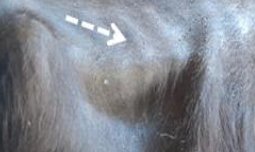	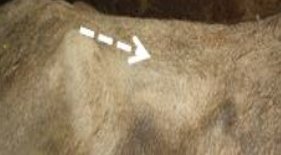	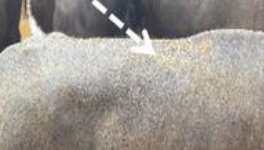
Hip bones (HB)	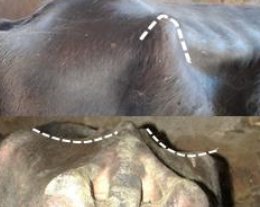	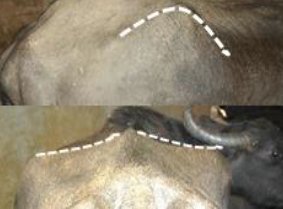	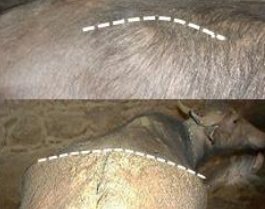
Rump (RP)	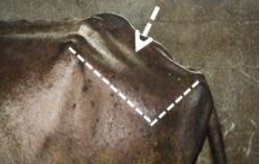	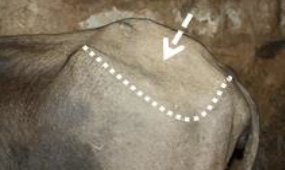	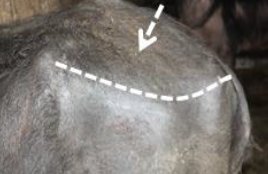
Pin bone (PB)	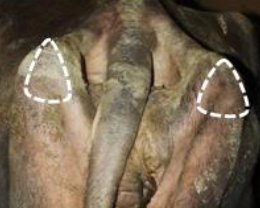	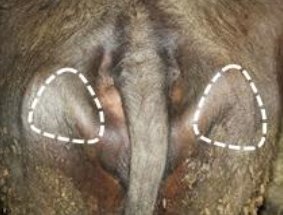	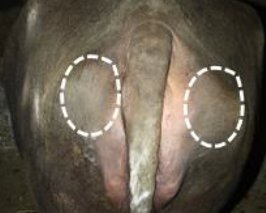

**Table 1:** Continued.

Variables	1–3 (thin)	4–6 (moderate)	7–9 (fat)
Tailhead (TL)	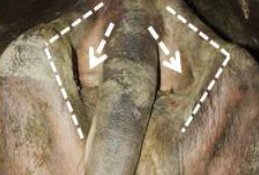	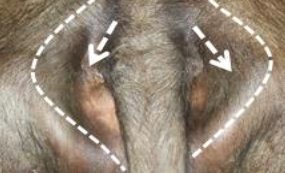	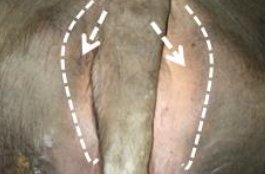
Thigh (TH)	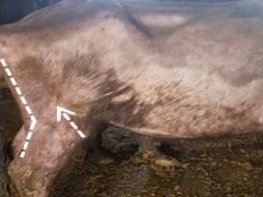	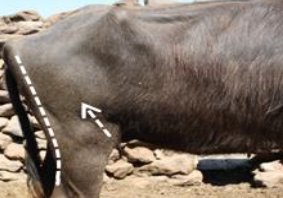	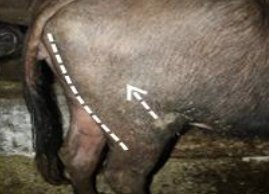

## 3 Results

BB has the highest average with 6.12 points, followed by RB (5.73), RP (5.69), and both SR and HB (5.65). TL has the lowest average, with 4.92 points. These results show that BB receives the most positive scores for the evaluated criteria, while TL receives the least positive scores. Examining the minimum and maximum values, all variables fall within similar ranges. The lowest minimum value is 2.50 for TL, and the highest maximum value is 8.50 for both BB and RB. This indicates that some variables have a wider range of scores (Table 2).

**Table 2:** Descriptive statistics of body condition variables ( n=614 ).

Variables	Mean	Min.	Max.	SD	Variance
BB	6.12	4.00	8.50	0.94	0.88
RB	5.73	3.50	8.50	0.97	0.95
SR	5.65	3.50	8.00	0.95	0.90
HB	5.65	3.00	8.00	0.84	0.71
RP	5.69	3.00	8.00	0.79	0.63
PB	5.25	3.00	8.00	0.72	0.52
TL	4.92	2.50	8.00	0.82	0.67
TH	5.10	3.00	8.00	0.79	0.62
Overall	5.51	3.63	7.81	0.69	0.48

BB = backbone; RB = ribs; SR = short ribs; HB = hip bones; RP = rump; PB = pin bone; TL = tailhead; TH = thigh; SD: standard deviation.

When examining the standard deviation and variance values, the highest standard deviation is for BB (0.94) and the lowest is for PB (0.72). Overall, the standard deviation and variance values for all variables are moderate, indicating that the data do not show substantial fluctuations. However, variables with higher standard deviations, such as BB and RB, have a more heterogeneous assessment distribution. The overall mean value is 5.51, indicating that the variables are generally assessed at a medium-high level. The overall standard deviation of 0.69 and variance of 0.48 indicate that the data have a consistent distribution, far from extreme outliers (Table 2).

The Kaiser–Meyer–Olkin (KMO) measure of sampling adequacy was 0.892. A KMO value between 0.80 and 0.90 indicates that the data are very well suited for factor analysis (Kaiser, 1974). Bartlett's test of sphericity was significant ( $\chi^{2}=3896.441$ , df = 28, p<0.001 ), indicating sufficient correlation among the variables to justify factor analysis (Bartlett, 1954). These results show that the dataset provides a strong statistical basis for factor analysis (Table 3).

**Table 3:** KMO and test results for data.

Kaiser–Meyer–Olkin measure		0.892
of sampling adequacy		
Bartlett's test	Approx. chi-square	3896.441
of sphericity	Df	28
	Sig.	.000

Df = degrees of freedom, Sig. = the p value indicating whether the test result is statistically significant.

It shows how much of each variable's variance is explained by the factors. In the Initial column, the value for all variables is 1.00, indicating that before factor analysis, the total variance of each variable is assumed to be entirely due to itself. The values in the Extraction column represent the proportion of variance explained by the factors identified in the factor analysis.

According to the results, the extent to which the factor structure explains the variables range from 0.725 to 0.859. In the literature, common variance values above 0.50 are considered acceptable. Therefore, the high values obtained in this study indicate that the factor analysis is reliable and that the variables are strongly related to the factor structure (Table 4).

**Table 4:** Commonality values in factor analysis.

Variables	Initial	Extraction
BB	1.000	0.763
RB	1.000	0.824
SR	1.000	0.837
HB	1.000	0.725
RP	1.000	0.784
PB	1.000	0.859
TL	1.000	0.848
TH	1.000	0.732

BB = backbone, RB = ribs; SR = short ribs; HB = hip bones; RP = rump; PB = pin bone; TL = tailhead; TH = thigh; SD = standard deviation.

In the factor analysis, the first factor (BB) has an eigenvalue of 5.26 and explains 65.70 % of the total variance. The second factor (RB) has an eigenvalue of 1.12 and explains 13.96 % of the total variance. According to the Kaiser criterion, these two factors – both with eigenvalues above 1 – together explain 79.67 % of the total variance. This indicates that the scale has a two-factor structure.

After rotation, the first factor explains 40.28 % of the total variance and the second factor explains 39.38 %. This indicates that the variance is distributed more evenly among the factors and that the interpretability of the resulting structure has increased. Other factors with eigenvalues below 1 were excluded from the analysis in accordance with the Kaiser criterion (Table 5).

**Table 5:** Total variance explained.

Component	Initial eigenvalues			Extraction sums of squared loadings			Rotation sums of squared loadings		
	Total	% of	Cumulative	Total	% of	Cumulative	Total	% of	Cumulative
		variance	%		variance	%		variance	%
BB	5.256	65.703	65.703	5.256	65.703	65.703	3.223	40.283	40.283
RB	1.117	13.963	79.666	1.117	13.963	79.666	3.151	39.384	79.666
SR	.435	5.435	85.102						
HB	.320	4.005	89.107						
RP	.296	3.699	92.806						
PB	.221	2.767	95.573						
TL	.188	2.356	97.929						
TH	.166	2.071	100.000						

BB = backbone, RB = ribs; SR = short ribs; HB = hip bones; RP = rump; PB = pin bone; TL = tailhead; TH = thigh.

When examining the scree plot, the eigenvalue of the first factor is approximately 5.2 and is clearly separated from the others. The eigenvalue of the second factor is slightly above 1 and, although its contribution is more limited, it is still considered meaningful. The graph shows that after the second factor, the curve flattens significantly, indicating that the eigenvalues decrease rapidly. According to Cattell's scree test, this suggests that including two factors in the analysis is appropriate. Therefore, both the eigenvalue criterion (Kaiser criterion, eigenvalue > 1) and the scree plot evaluation indicate that the scale has a two-factor structure (Fig. 1).

**Figure 1:**
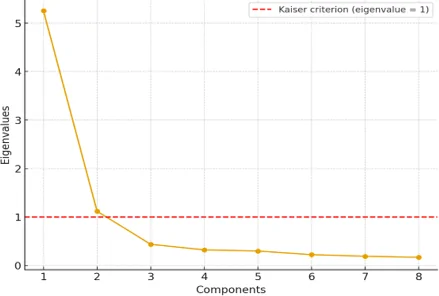
Distribution of eigenvalues.

In the study, variables related to body condition were grouped into two components based on principal component analysis. The extracted components were examined to identify the dimensions in which the variables were concentrated.

The component analysis revealed the fundamental dimensional structures underlying the physical fitness variables. The first component (PC1: vertebral and posterior body regions) had high positive loadings on variables such as RP (0.884), HB (0.847), BB (0.809), TL (0.797), SR (0.788), TH (0.787), PB (0.786), and RB (0.780). These results indicate that PC1 is a dominant factor representing the general dimensional size of the skeletal system. The high factor loadings indicate that this component is strongly related to the overall metric size of body condition.

The second component (PC2: pelvic and lower-body regions) showed a lower level and a heterogeneous loading distribution. In this component, the variables TL (0.462) and PB (0.492) had notably positive loadings, while the other variables had low or negative loadings. This suggests that PC2 represents a secondary level of variation among body condition variables and reflects different structural dimensions, particularly those related to regions with lower-body condition (Table 6).

**Table 6:** Component matrix.

Variables	Component	
	PC1	PC2
RP	0.884	0.051
HB	0.847	- 0.081
BB	0.809	- 0.329
TL	0.797	0.462
SR	0.788	- 0.465
TH	0.787	0.336
PB	0.786	0.492
RB	0.780	- 0.464

PC1 = vertebral and posterior body regions; PC2 = pelvic and lower-body regions; BB = backbone, RB = ribs; SR = short ribs; HB = hip bones; RP = rump; PB = pin bone; TL = tailhead; TH = thigh.

In general, PC1 has very high explanatory power, with most variables contributing high loadings to this component. In contrast, PC2 represents more limited and specific variation. The results show that body measurements cluster primarily around a single principal dimension (PC1), and some anatomical regions exhibit secondary variation captured by PC2.

The loadings of the examined features on the PC1 and PC2 axes are shown in the PCA biplot. The graph reveals two main patterns, with vectors indicating feature directions and strengths. All morphological features have high positive loadings on the PC1 axis, indicating that PC1 reflects the common variance in overall body size and skeletal structure. The RP, HB, and BB features contribute most to the PC1 direction and therefore most strongly represent this component (Fig. 2).

**Figure 2:**
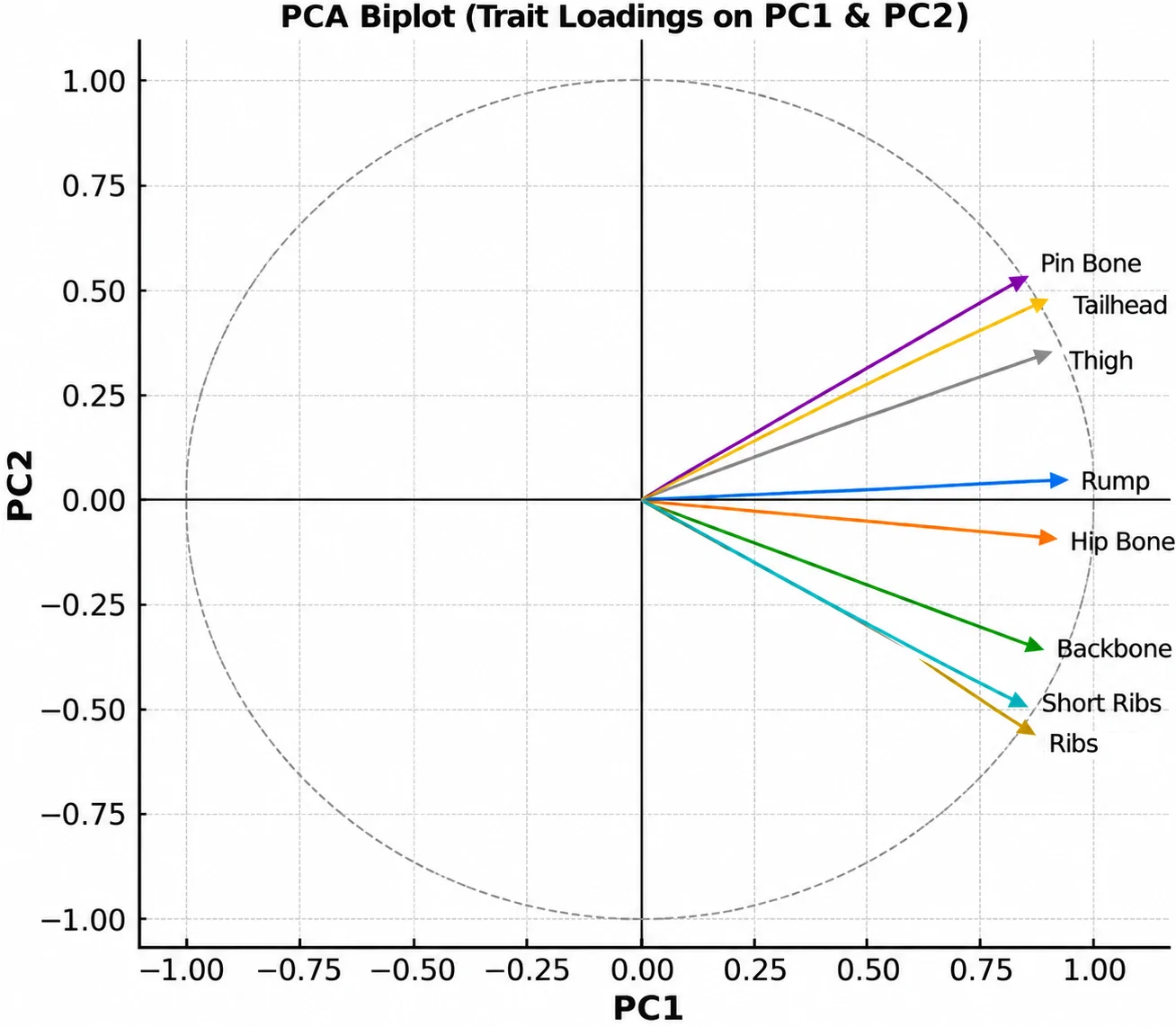
Component characteristic loads.

On the PC2 axis, the characteristics are divided into two groups. PB, TL, and TH have positive loadings, whereas SR, RB, and BB have negative loadings. This opposite orientation indicates that PC2 represents the difference between rear body structure and body depth. In conclusion, the biplot graph shows that variation in morphological features can be explained by two principal dimensions: vertebral and posterior body regions (PC1) and pelvic and lower-body regions (PC2) (Fig. 2).

Varimax rotation was applied to enable a more meaningful interpretation of the factors. After rotation, the first factor explains 40.283 % of the variance, and the second factor explains 39.384 %. This result indicates a more balanced distribution of variance between the factors.

The first factor includes the variables SR (0.888), RB (0.882), BB (0.808), HB (0.661), and RP (0.595). This factor represents the body's supporting skeletal structure and is named the vertebral–posterior body factor. The second factor includes the variables PB (0.902), TL (0.888), TH (0.791), RP (0.656), and HB (0.536). Because this factor relates to the pelvic region and lower-body structures, it is named the pelvic–lower-body skeletal factor (Table 7).

**Table 7:** Rotated component matrix.

Variables	Component	
	PC1	PC2
SR	0.888	0.220
RB	0.882	0.216
BB	0.808	0.333
HB	0.661	0.536
PB	0.215	0.902
TL	0.245	0.888
TH	0.326	0.791
RP	0.595	0.656

PC1 = vertebral and posterior body regions; PC2 = pelvic and lower-body regions; BB = backbone, RB = ribs; SR = short ribs; HB = hip bones; RP = rump; PB = pin bone; TL = tailhead; TH = thigh.

The variables for the pelvic bone and hip region showed significant loadings on both factors. This can be attributed to the anatomical characteristics of these structures, which are related to both the trunk and the pelvic system. As a result, the factor analysis revealed that the scale has a two-dimensional structure, and these two dimensions explain a high level of variance. This finding indicates that the scale has strong construct validity.

## 4 Discussion

In this study, we evaluated body measurement variables to determine body condition in Anatolian water buffaloes and used factor analysis to examine the underlying structure of these measurements. Descriptive statistics show that the BB variable has the highest mean score, whereas the TL variable has the lowest mean. This suggests that the spine region is a primary determinant of body condition, whereas the tail region is more variable and yields lower scores. Standard deviation and variance values indicate a moderate level of variation among the variables overall and show that the dataset has a consistent distribution with few extreme outliers (Field, 2013).

Preliminary tests for factor analysis (KMO = 0.892; Bartlett's $\chi^{2}=3896.441$ , p<0.001 ) indicated that the data were suitable for factor analysis. This result supports the presence of meaningful correlations among the variables and shows that the measurements can be explained by common factors. The common variance values, ranging from 0.725 to 0.859, indicate that most variables have a strong relationship with the factor structure and that the analysis is highly reliable (Hair et al., 2019).

In the present study, PCA was applied to eight body condition traits in Anatolian water buffalo cows to identify the underlying structural dimensions of body condition. The results showed that two principal components effectively summarized body condition, explaining 79.67 % of the total variance. This relatively high proportion of explained variance indicates a strong internal structure and suggests that the selected condition traits are highly interrelated and biologically meaningful.

The first principal component (PC1), which accounted for 65.70 % of the total variance before rotation, was characterized by high positive loadings for all body condition traits, particularly RP, HB, BB, RB, and SR. This component represents a general body condition dimension associated with vertebral and posterior body regions, and reflects the overall level of fat deposition and skeletal coverage. Similar findings have been reported in previous water buffalo studies, where the first principal component was interpreted as a general body size or body condition factor (Vohra et al., 2015; Putra and Hilmawan, 2022). The dominance of PC1 indicates that variation in body condition among Anatolian water buffalo cows is driven primarily by changes in overall energy reserves rather than variation in isolated anatomical regions.

The second principal component (PC2), which explained 13.96 % of the total variance, was primarily associated with pelvic and lower-body regions, particularly PB, TL, and TH. Although its contribution to the total variance was lower, PC2 captured region-specific variation not fully explained by PC1. After varimax rotation, the variance explained by the two components became more balanced, improving interpretability and supporting the presence of two distinct yet complementary structural dimensions of body condition. The cross-loadings observed for pelvic- and hip-related traits are anatomically expected, as these regions are functionally connected to both the trunk and hindquarters. Similar cross-loading patterns have been reported in morphometric PCA studies of buffalo, cattle, sheep, and goats, reflecting the shared anatomical and functional roles of these regions (Salako, 2006; Khargharia et al., 2015; Karacaören and Kadarmideen, 2008).

The adequacy of the data for factor analysis was supported by a high KMO value (0.892) and a highly significant Bartlett's test of sphericity, indicating strong correlations among the variables. Communalities ranged from 0.725 to 0.859, suggesting that a substantial proportion of variance in each trait was explained by the extracted components. According to multivariate analysis guidelines, these values indicate a reliable, well-defined factor structure (Hair et al., 2019). Compared with previous studies that used a larger number of morphometric variables, the present study achieved a higher proportion of explained variance with fewer variables, likely because it used body condition scores, which directly reflect fat reserves rather than linear body dimensions.

Compared with PCA studies in dairy cattle, where body condition score is often analyzed together with milk yield, live weight, and feed intake, the current study provides a more focused evaluation of body condition itself (Karacaören and Kadarmideen, 2008). This focused approach allows clearer interpretation of the structural components underlying body condition and enhances its practical applicability for herd management. In small ruminants such as sheep and goats, PCA studies generally report lower explained variance and a greater number of components, reflecting more heterogeneous body conformation and fat distribution patterns (Salako, 2006; Khargharia et al., 2015). By contrast, the more homogeneous fat deposition patterns in buffaloes may account for the strong, compact two-component structure observed in this study.

Overall, the findings indicate that body condition in Anatolian water buffaloes can be reliably described by two main structural dimensions: a general body condition dimension related to the vertebral and posterior body regions, and a secondary dimension associated with the pelvic and lower-body regions. This two-dimensional structure provides a scientifically grounded, objective framework for evaluating body condition, with potential applications in breeding programs, nutritional management, and health monitoring. Future studies that incorporate age, lactation stage, reproductive status, and performance traits such as milk yield will further enhance the biological interpretation and practical relevance of these components.

## 5 Conclusion

In conclusion, principal component analysis (PCA) identified the underlying structural dimensions of eight body condition traits evaluated in Anatolian water buffaloes. The results showed that the traits were not independent but could be grouped into a smaller number of principal components representing common patterns of body condition variation. The first principal components accounted for a substantial proportion of the total variation, indicating that PCA can effectively summarize the information contained in multiple correlated body condition traits.

These findings provide a more concise representation of body condition structure and may facilitate the interpretation of phenotypic variation among Anatolian water buffaloes. Although the present study did not directly evaluate the implications of these components for herd management, reproductive performance, or breeding decisions, the principal components identified provide a useful basis for further research on the potential applications of multivariate body condition assessment in these areas.

## Data Availability

The data were obtained from a project conducted under the Scientific Research Projects Coordination Office (BAP) at Muş Alparslan University. In accordance with the university's institutional data-sharing policy, the data may be obtained from the corresponding author upon reasonable request.
